# A Mapping of Operative Heterogeneity in Robotic Splenic Flexure Cancer Surgery, Focusing on Vascular Ligation and Reconstructive Strategy

**DOI:** 10.3390/cancers18091490

**Published:** 2026-05-06

**Authors:** Mohit Satish Gupta, Georgios Kyriakopoulos, Sina Hossaini, Mick Harper, Gerald David, Guglielmo Piozzi, Filippos Sagias, John Conti, Jim Khan

**Affiliations:** 1 Portsmouth Hospitals University NHS Trust, Portsmouth PO3 6LY, UK; georgios.kyriakopoulos@nhs.net (G.K.); jim.khan2@nhs.net (J.K.); 2 School of Health and Care Professions, Faculty of Science and Health, University of Portsmouth, Portsmouth PO1 2UP, UK; mick.harper@port.ac.uk

**Keywords:** splenic flexure cancer, robotic colectomy, vascular ligation, intracorporeal anastomosis, mesocolic excision, scoping review

## Abstract

**Background:** Splenic flexure cancers represent a small but anatomically complex subset of colorectal malignancies, characterised by variable vascular supply and lymphatic drainage. This has led to ongoing uncertainty regarding the optimal extent of resection and level of vascular ligation. With increasing adoption of robotic surgery, a range of operative approaches has been described, although these remain inconsistently reported and poorly synthesised. **Aim:** We aimed to map operative heterogeneity in robotic splenic flexure cancer surgery, focusing on resection extent, vascular ligation, reconstruction strategy, and technical configuration. **Methods:** A structured scoping review was performed in accordance with PRISMA-ScR guidelines. MEDLINE (PubMed) and Embase (Ovid) were searched from inception to January 2026. Studies reporting robotic resection for primary splenic flexure colon cancer with extractable operative detail were included. Data were synthesised descriptively and grouped into three tiers based on study design: comparative cohorts, case series, and technical reports. **Results:** Sixteen studies comprising 97 robotic resections were included. Three were comparative cohort studies; two were case series, and eleven were single-case technical reports. Comparative studies consistently described a flexure-directed segmental resection with preservation of the inferior mesenteric artery and division of the left colic artery. In contrast, there was greater variability in management of the middle colic branches and inferior mesenteric vein. Reconstruction strategy differed across tiers, with extracorporeal anastomosis more commonly reported in comparative cohorts, while intracorporeal techniques were frequently described in technical series. Anastomotic configuration, specimen extraction, and use of indocyanine green fluorescence varied widely. Terminology relating to complete mesocolic excision and D2/D3 lymphadenectomy was inconsistent and often poorly defined. **Conclusions:** Robotic splenic flexure surgery shows broad agreement in overall resection strategy but considerable variability in vascular control, reconstruction, and technical execution. This likely reflects differences in reporting rather than clear differences in oncological intent. A more consistent and structured operative description is needed to allow meaningful comparison and to support the development of evidence-based approaches in this technically demanding area.

## Simple Summary

Robotic surgery is increasingly used to treat splenic flexure cancers, where dual blood supply from the middle and left colic arteries and variable venous drainage make standardisation of operative technique particularly challenging. Despite a growing literature, fundamental aspects of the operation—including the level of vascular ligation, the extent of lymphadenectomy along the inferior mesenteric and middle colic axes, and the configuration of intracorporeal reconstruction—remain inconsistently reported, limiting meaningful comparison between studies and between centres. In this structured scoping review of 16 studies and 97 robotic resections, we found broad convergence at the level of overall resection philosophy but persistent variability in vascular control, central lymphadenectomy at the IMA root, and reconstructive technique. Much of this apparent variation reflects differences in how operations are reported rather than genuine differences in surgical intent, with critical oncological steps such as peri-IMA nodal clearance documented in only a minority of studies. To address this, we propose a structured operative reporting template, based on a macro–meso–micro analytical framework and a defined operative lexicon, that future studies can adopt to support consistent description, meaningful comparison, and the development of evidence-based standards in this technically demanding area of colorectal oncology.

## 1. Introduction

Splenic flexure cancers account for approximately 2–8% of colonic malignancies and represent a distinct anatomical and surgical entity within colorectal cancer management [1]. Positioned at the junction of the transverse and descending colon, tumours in this region demonstrate variable arterial supply from branches of the middle colic artery (MCA), left colic artery (LCA), or both, with corresponding complexity in venous drainage and lymphatic pathways [2,3]. This dual vascularisation has historically raised uncertainty regarding the optimal extent of resection and level of vascular ligation necessary to achieve oncological adequacy. In addition to vascular complexity, operative strategy at the splenic flexure is influenced by variation in omental management and entry into the lesser sac. Division of the gastrocolic ligament and mobilisation of the greater omentum may be performed either as an omentum-preserving dissection along the transverse mesocolon or as an en bloc omentectomy when oncologically indicated [4]. These variations reflect differing approaches to splenic flexure mobilisation and may influence exposure of the middle colic vessels and pancreatic border. The concept of complete mesocolic excision (CME) with central vascular ligation has influenced contemporary surgical philosophy by emphasising dissection along embryological planes, en bloc mesocolic resection, and central ligation of feeding vessels to maximise lymph node harvest and oncological clearance [4,5]. However, application of CME principles to splenic flexure cancers remains inconsistently described, particularly with respect to IMA ligation level, LCA division, and management of the MCA branches [6,7]. Variability in terminology, including inconsistent use of D2 and D3 classifications, further complicates interpretation of the literature [8]. The increasing adoption of robotic colorectal surgery introduces additional technical variables that may influence operative strategy, including enhanced three-dimensional visualisation, wristed instrumentation, and improved ergonomics which facilitate precise vascular dissection and intracorporeal reconstruction [9,10]. Preoperative vascular mapping using contrast-enhanced CT with three-dimensional reconstruction has been increasingly utilised to define individual arterial and venous anatomy and to support targeted vessel dissection in splenic flexure tumours, where variable middle colic and left colic branching patterns may influence operative strategy [11]. At the same time, robotic platforms expand the range of technically feasible approaches, including selective branch ligation, intracorporeal anastomosis, fluorescence-guided perfusion assessment, and varied docking configurations.

Importantly, these platform-specific capabilities may alter the balance between extended and segmental resection strategies and may amplify variation in operative technique compared with earlier laparoscopic series. Despite growing publication of robotic splenic flexure resections, existing studies predominantly focus on short-term perioperative outcomes, conversion rates, and length of stay. Operative technique is often described variably and without systematic characterisation of vascular ligation strategy, mesocolic dissection boundaries, or reconstructive configuration. To date, no structured synthesis has specifically mapped operative heterogeneity within robotic splenic flexure cancer surgery. The aim of this structured scoping review is to systematically map and characterise operative heterogeneity in robotic splenic flexure colon cancer surgery. Specifically, this review examines variation in:

- Extent of resection.
- Arterial ligation strategy.
- Inferior mesenteric vein management.
- Reconstruction approach and configuration.
- Platform utilisation and technical modifiers.

By identifying patterns of convergence, divergence, and reporting inconsistency, this synthesis seeks to inform future surgical standardisation approaches and facilitate more meaningful comparative research in robotic colorectal oncology.

## 2. Methods

### 2.1. Study Design

This structured scoping review was conducted in accordance with the Preferred Reporting Items for Systematic Reviews and Meta-Analyses extension for Scoping Reviews (PRISMA-ScR).

The objective was to systematically map heterogeneity in robotic splenic flexure colon cancer surgery, with specific focus on:

- Extent of resection strategy.
- Arterial ligation pattern (IMA, LCA, MCA).
- Inferior mesenteric vein management.
- Reconstruction approach and configuration.
- Platform utilisation and docking strategy.

This review was designed as a structured synthesis to map and characterise operative heterogeneity in robotic splenic flexure colon cancer surgery, rather than to evaluate comparative effectiveness.

Two reviewers independently performed title, abstract, and full-text screening against predefined eligibility criteria. Discrepancies were resolved through discussion and consensus.

### 2.2. Review Question and Framework

The review was structured around the following question:

What operative strategies are reported in robotic splenic flexure colon cancer surgery, and how do these vary with respect to resection extent, vascular ligation, reconstruction, and technical configuration?

The framework was defined as:

**Population:** Adult patients undergoing robotic resection for primary splenic flexure colon adenocarcinoma.

**Concept:** Operative technique, including resection extent, vascular ligation pattern, venous management, reconstruction strategy, and platform configuration.

**Context:** Elective robotic colorectal surgery.

Because the objective was to map heterogeneity rather than evaluate comparative effectiveness, no formal comparator or outcome hierarchy was prespecified.

The protocol for this structured scoping review was developed a priori and registered retrospectively on the Open Science Framework (OSF) (https://osf.io/7p2na; registered 28 February 2026).

### 2.3. Eligibility Criteria

#### 2.3.1. Inclusion Criteria

Studies were eligible if they met all of the following criteria:

- Adult patients undergoing elective robotic or robot-assisted resection for primary splenic flexure adenocarcinoma.
- Extractable operative detail in at least one of the following domains:
- Arterial ligation strategy (IMA level, LCA division, MCA management).
- Inferior mesenteric vein handling.
- Extent of resection.
- Reconstruction approach (intracorporeal vs. extracorporeal; configuration).
- Docking configuration or operative sequencing.
- Original research articles, including:
- Comparative cohort studies.
- Case series.
- Case reports.
- Video-based technical publications.

#### 2.3.2. Exclusion Criteria

- Open or purely laparoscopic surgery.
- Rectal, sigmoid, or non-splenic flexure tumours.
- Mixed colorectal cohorts where splenic flexure cases were not separable.
- Review articles, editorials, and conference abstracts.
- Full text unavailable after reasonable retrieval attempts.
- English language only.

### 2.4. Information Sources and Search Strategy

A structured search was performed in:

- PubMed (MEDLINE).
- Embase (Ovid).

A structured search of PubMed (MEDLINE) and Embase (Ovid) was conducted from database inception to 15 February 2026. The search strategy combined terms relating to splenic flexure, robotic surgery, and colon cancer, with no date restrictions applied. Full search strategies for each database are provided in Supplementary Material S1.

### 2.5. Study Selection

All records were exported in Research Information Systems (RIS) format and imported into Rayyan for deduplication and screening.

A total of 102 records were identified across the databases, including 65 from PubMed and 37 from Embase (Ovid). After removal of duplicates (*n* = 18), 84 unique records underwent title and abstract screening by the two reviewers, and sixty-one records were excluded at this stage for not meeting the inclusion criteria.

Twenty-three studies were deemed potentially eligible and underwent further evaluation. Of these, five records were conference abstracts only and full texts were unavailable despite reasonable retrieval efforts. One record was identified as a duplicate at full-text stage.

**Figure 1:**
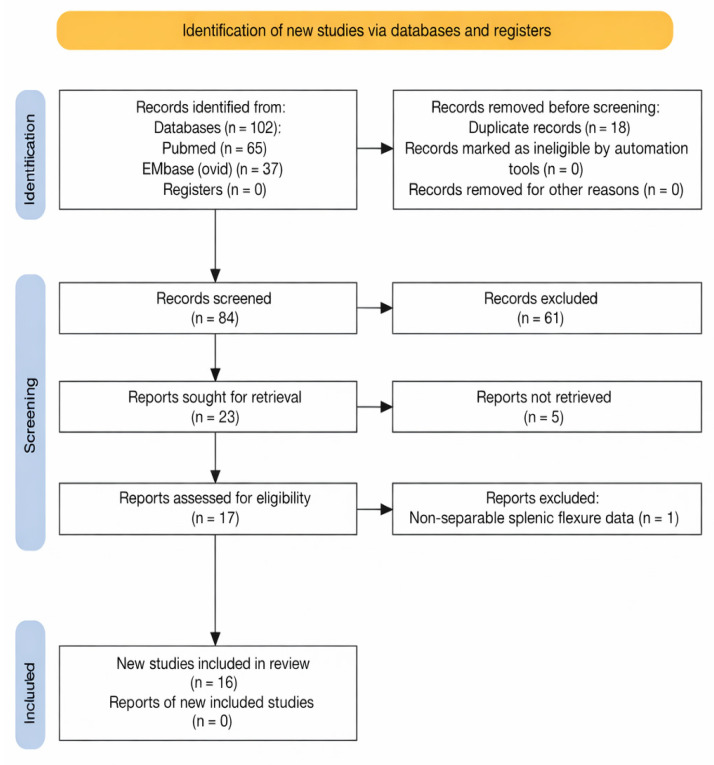
PRISMA 2020 flow diagram of study selection. Flow diagram demonstrating identification, screening, eligibility assessment, and inclusion of studies evaluating robotic splenic flexure cancer surgery.

Seventeen full-text articles were assessed for eligibility. One study was excluded after full-text review due to non-separable splenic flexure data, with sixteen studies included in the final analysis. Study selection is illustrated in the PRISMA flow diagram (Figure 1).

### 2.6. Study Stratification (Tier Classification)

Included studies were stratified according to study design to contextualise level of evidence and reporting maturity. Studies were categorised into three predefined tiers:

**Tier 1:** Comparative cohort studies.

**Tier 2:** Multi-patient case series.

**Tier 3:** Single case reports, technical notes, and video vignettes.

This classification was applied following inclusion and was used to structure descriptive synthesis across operative domains.

### 2.7. Data Extraction

Data were extracted using a predefined operative mapping framework developed a priori. Extracted variables included study characteristics (country, design, platform, and follow-up), extent of resection terminology, arterial ligation pattern (IMA, LCA, and MCA), inferior mesenteric vein management, reconstruction approach and configuration, extraction site, fluorescence use, and docking strategy.

Terminology was recorded verbatim where possible. However, where operative descriptions were ambiguous, interpretation was conservative and limited to explicitly reported anatomical detail.

### 2.8. Data Synthesis

Given substantial heterogeneity in study design, reporting structure, and outcome measures, quantitative meta-analysis was not performed. Findings were synthesised descriptively and organised by operative domain. Comparative interpretation across tiers was undertaken to evaluate patterns of reporting and technical convergence.

Formal risk-of-bias assessment was not conducted, as the objective was technical mapping rather than effect estimation.

## 3. Results

### 3.1. Study Selection and Evidence Landscape

A total of 16 studies met inclusion criteria for this structured scoping review (Table 1, Table 2 and Table 3). Studies were stratified according to design and level of evidence to contextualise the maturity of the available literature.

Three studies were comparative cohort analyses (Tier 1), including two retrospective comparative cohorts and one propensity score-matched study (Table 1). Collectively, these reported 71 robotic splenic flexure cancer resections and represented the highest level of comparative evidence identified.

Two studies were case series (Tier 2), comprising one retrospective cohort and one prospective case series (Table 2). These contributed 15 additional robotic splenic flexure resections.

The remaining 11 studies were technical notes or video vignettes (Tier 3), each describing a single-case operative demonstration (Table 3). These contributed 11 individual cases and were primarily descriptive, without structured comparative methodology.

In total, 97 robotic splenic flexure cancer resections were identified across all tiers.

Only five of sixteen studies reported more than one patient in a structured cohort format (Tiers 1 and 2). No randomised trials or multicentre prospective registries specific to robotic splenic flexure cancer surgery were identified.

Follow-up duration and oncologic endpoints were variably reported, particularly among Tier 3 publications. Terminology describing extent of resection, vascular ligation strategy, and reconstruction technique was not uniformly defined across studies.

### 3.2. Extent of Resection Strategy

Across the included studies, resection for splenic flexure cancer was most commonly described as a segmental or splenic flexure-focused colectomy (Table 4) and terminology varied between publications.

In Tier 1 and Tier 2 studies, the operative extent was consistently limited to the splenic flexure region and described as segmental resection, splenic flexure resection/colectomy, or partial left colectomy. Within the included comparative studies, both robotic and laparoscopic cohorts predominantly underwent directed segmental or left colectomy resections, and no study described routine extended right hemicolectomy as the standard approach for isolated splenic flexure malignancy.

The term “left colectomy” was used in several studies, particularly where resection included the distal transverse and proximal descending colon. However, operative descriptions in these reports remained consistent with a flexure-centred resection strategy rather than a formal extended hemicolectomy.

Explicit reference to complete mesocolic excision (CME) was reported in a subset of studies, including selected Tier 1 cohorts and multiple Tier 3 technical publications (Table 2). However, CME terminology was not consistently accompanied by standardised descriptors of central vascular ligation or defined lymphadenectomy boundaries.

Only one comparative study explicitly referenced D3 lymph node dissection. In most publications, lymphadenectomy was described qualitatively rather than using a consistent oncologic classification framework.

Overall, while convergence toward flexure-directed resection was observed, definitions of operative extent and oncologic classification (CME/D3) were not uniformly reported across tiers (Table 2).

**Evidence Tier Key**

Tier 1—Comparative cohort.

Tier 2—Multi-patient case series.

Tier 3—Single case report/video/technical note.

### 3.3. Vascular Ligation Strategy

Vascular management demonstrated substantial heterogeneity across studies and evidence tiers (Table 5).

#### 3.3.1. IMA Management

Among Tier 1 comparative studies, a low-tie strategy was consistently described, with preservation of the main inferior mesenteric artery (IMA) trunk and superior rectal artery. In all three comparative cohorts (Kim, Sugishita, Zang), the left colic artery (LCA) was divided while the IMA trunk was preserved. None of the comparative studies reported routine high ligation of the IMA [12,13,14]. Tier 2 case series largely reflected the Tier 1 low-tie IMA preservation strategy. Monsellato et al. [15] described preservation of the IMA trunk with division of the LCA at its origin in most cases, including selective branch preservation in one patient. Carannante et al. [16] did not provide sufficient detail to determine the level of IMA ligation.

In Tier 3 technical reports, greater variability was observed. Most described division of the LCA with preservation of the IMA trunk (Aghayeva, Benlice, Bourla, Carrier, Milone, Quezada-Diaz). [17,18,19,23,25,26] Erozkan et al. [21] described ligation of the inferior mesenteric artery and vein without clarification of trunk preservation. Several reports did not explicitly define the level of IMA control.

#### 3.3.2. Left Colic and Middle Colic Management

Division of the LCA at its origin from the IMA was the most frequently described strategy across tiers and was consistently reported in all Tier 1 studies (Table 3) [12,13,14].

Management of the middle colic artery (MCA) demonstrated greater variability. In comparative cohorts, division of the left branch of the MCA was commonly described [12,13,14]. Similar branch division was reported in several Tier 3 publications (Bourla, Carrier, Monsellato video, Quezada-Diaz) [19,23,24,25]. One report (Aghayeva) described ligation of the middle colic artery and vein without specifying branch versus trunk division [17]. Several studies did not explicitly describe MCA management. In addition, selective branch preservation strategies were described in isolated reports, including skeletonization with division of only the ascending branch of the LCA [24].

#### 3.3.3. Inferior Mesentric Vein Management

Management of the inferior mesenteric vein (IMV) was inconsistently reported. In Tier 1 studies, the IMV was typically divided at the inferior border of the pancreas. This level was also described in several Tier 2 and Tier 3 studies (Kim, Zang, Monsellato case series, Aghayeva, Benlice, Bourla) [12,13,15,17,18,26].

Alternative approaches were reported; for example, Carrier et al. described ligation near the ligament of Treitz. Whilst Milone et al. described division at the root [19] and Quezada-Diaz et al. described preservation of the IMV to maintain venous drainage of the distal colon [25]. The level of IMV control was frequently mentioned descriptively but without consistent anatomical classification.

#### 3.3.4. Central Vascular Ligation and Oncologic Classification

Explicit terminology regarding central vascular ligation or D3 dissection was inconsistently applied (Table 5). Sugishita et al. referenced D3 lymph node dissection [14]. Kim and Zang described adherence to principles of complete mesocolectomy [12,13]. Several Tier 3 reports referenced complete mesocolic excision; however, detailed anatomical definitions of central ligation were often not specified [11,17,18,19,20,23,24].

In many technical reports, lymphadenectomy was described qualitatively without reference to a formal oncologic classification system.

Explicit reporting of central lymphadenectomy surrounding the IMA, independent of the decision to preserve or ligate the IMA trunk, was inconsistent across included studies. Five of sixteen studies (31%) described dissection of the nodal tissue at the IMA root or origin of the LCA with varying levels of anatomical precision. Bourla et al. described lymph node dissection commencing along the IMA and continuing to the take-off of the LCA prior to vessel division. Monsellato et al. (Minerva Surgery) explicitly described complete lymphadenectomy at the origin of the IMA as a discrete step preceding LCA division. Monsellato et al. (video vignette) similarly documented lymphadenectomy of the IMA alongside skeletonisation of the LCA. Zang et al. described dissection of the No. 253 lymph node station at the IMA root, in accordance with Japanese anatomical classification. Sugishita et al. reported D3 dissection in 34.4% of cases without providing a descriptive account of the dissection steps. In the remaining eleven studies (69%), nodal clearance at the IMA root was not explicitly described; several reports mentioned LCA division at its origin or made generic reference to complete mesocolic excision without specifying whether the peri-IMA nodal envelope was dissected and retrieved (Table 5).

This distinction is oncologically significant. IMA trunk preservation is compatible with D3 nodal clearance only if the peri-IMA nodal envelope at the LCA origin is dissected. Division of the LCA without accompanying central nodal clearance represents a materially different oncological procedure, regardless of the label applied. The absence of this detail in the majority of included studies represents one of the most consequential reporting gaps identified in this review.

#### 3.3.5. Summary of Vascular Strategy

Across comparative cohorts, a consistent pattern of low-tie IMA management with division of the LCA and left branch of the MCA was observed [12,13,14]. Variation was present in IMV level, branch preservation strategies, and in the explicit definition of central vascular ligation, particularly within Tier 3 publications (Table 3). Reporting of lymphadenectomy was heterogeneous. Some studies explicitly described nodal dissection along the IMA or LCA or referenced CME/D3 principles, whereas others implied lymphadenectomy through vascular dissection without defining anatomical nodal boundaries.

### 3.4. Reconstruction Strategy

Reconstruction strategy varied across evidence tiers, including differences in anastomotic approach, configuration, extraction site, and use of fluorescence assessment (Table 6).

#### 3.4.1. Intracorporeal Versus Extracorporeal Anastomosis

In Tier 1 comparative studies, extracorporeal reconstruction predominated. Kim et al. and Zang et al. performed extracorporeal anastomosis in all robotic cases [12,13]. Sugishita et al. reported a mixed approach, with extracorporeal reconstruction in 62.5% and intracorporeal reconstruction in 34.4% of robotic cases [14].

In contrast, Tier 2 and Tier 3 publications more frequently described intracorporeal reconstruction. Both case series (Monsellato and Carannante) reported exclusively intracorporeal anastomosis [15,16]. Among Tier 3 technical reports, intracorporeal anastomosis was explicitly described in the majority of studies (Benlice, Bourla, Carrier, Da Silva, Iosa, Milone, Monsellato video, Quezada-Diaz) [18,19,20,22,23,24,25]. Lecot et al. described extracorporeal reconstruction [11].

#### 3.4.2. Anastomotic Configuration

**Figure 2:**
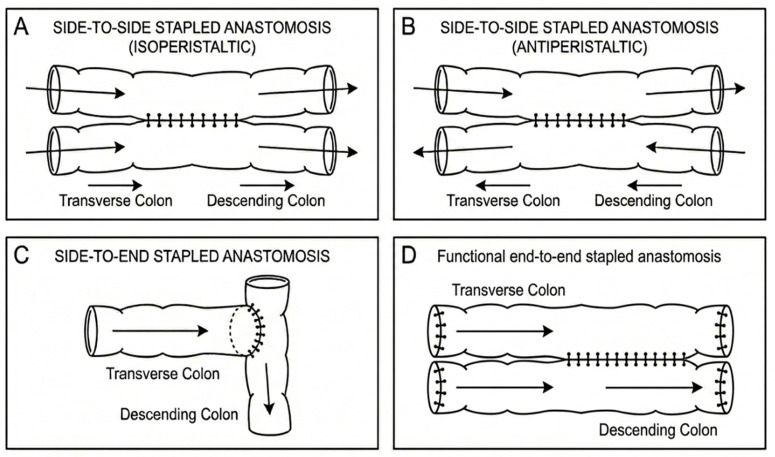
Anastomotic configurations following splenic flexure colectomy. Schematic illustration of commonly described reconstruction techniques after splenic flexure resection. (**A**) Isoperistaltic side-to-side stapled anastomosis. (**B**) Antiperistaltic side-to-side stapled anastomosis. (**C**) Side-to-end stapled anastomosis. (**D**) Functional end-to-end stapled anastomosis created by a side-to-side stapled anastomosis between two closed bowel ends. Arrows indicate direction of bowel peristalsis.

Across tiers, the most frequently described configuration was side-to-side stapled anastomosis (Table 4, Figure 2). Variation in orientation and technique was observed.

In Tier 1 studies, Kim et al. predominantly performed side-to-end stapled anastomosis [12]. Conversely, Sugishita et al. reported functional end-to-end anastomosis in 65.6% and overlap technique in 31.2% of cases [14] and Zang et al. described side-to-side anastomosis using a double-stapling technique [13].

Tier 2 and Tier 3 publications demonstrated additional configurational diversity. Isoperistaltic side-to-side anastomosis was described in several reports [23,25,26], whereas anisoperistaltic or antiperistaltic side-to-side configuration was reported in others [15,24]. Notably, only one study reported a manual end-to-end handsewn intracorporeal anastomosis [22].

#### 3.4.3. Extraction Site

Extraction site varied across tiers and was closely related to the method of reconstruction. Comparative studies performing extracorporeal anastomosis (ECA) typically reported transverse or epigastric midline mini-laparotomy incisions to allow exteriorisation of the bowel for reconstruction [12,13,14].

In contrast, studies describing intracorporeal anastomosis (ICA) more frequently utilised Pfannenstiel or suprapubic extraction sites, often achieved through extension of a robotic port site [15,16,20,23,25]. These approaches permitted specimen retrieval without the need for bowel exteriorisation and were typically associated with intracorporeal reconstruction techniques.

#### 3.4.4. Summary of Reconstruction Strategy

While side-to-side stapled anastomosis was the most frequently described technique, variation was observed in:

- Intracorporeal versus extracorporeal approach.
- Anastomotic orientation.
- Extraction site.
- Use of fluorescence guidance.

These variations were distributed differently across tiers, with intracorporeal reconstruction and Pfannenstiel extraction more commonly reported in Tier 2 and Tier 3 publications (Table 4).

### 3.5. Platform Variation and Technical Modifiers

Variation was observed in robotic platform utilisation, docking strategy, port configuration, fluorescence integration, and technique-specific adaptations (Table 4).

#### 3.5.1. Robotic Platform Utilisation

Across tiers, the da Vinci platform predominated, with the Xi system most frequently specified in recent publications.

In Tier 1 comparative studies, multiple da Vinci generations (Si, Xi, X) were reported [12,13,14]. One comparative cohort utilised the Si platform exclusively [13].

Among Tier 2 case series, both Si and Xi systems were reported in sequential adoption [15]. One study described use of the Hugo™ RAS modular robotic system, representing the only non-da Vinci platform within the included literature [16].

In Tier 3 technical reports, the da Vinci Xi platform was most commonly specified [18,19,20,21,23]. Several video publications did not explicitly state the robotic system utilised.

#### 3.5.2. Docking Strategy and Port Configuration

Docking strategy was inconsistently reported. Among Tier 1 studies, only one comparative cohort explicitly described a single-docking technique [13]. Docking configuration was not clearly specified in the remaining comparative publications [12,14].

In Tier 2 publications, both multi-docking and single-docking strategies were described depending on platform generation [15]. Single docking was also reported with the Hugo™ system [16].

Tier 3 reports more frequently detailed docking configuration, including single-docking crossed-arm techniques and fully robotic single-docking approaches [21,25].

Port configuration demonstrated additional variability. A universal port placement strategy was described in one comparative study [12], while a suprapubic approach with Pfannenstiel-aligned robotic ports was reported in a technical publication [20]. Other reports described oblique alignment or did not provide detailed port mapping.

#### 3.5.3. Fluorescence (ICG) Integration

Use of indocyanine green (ICG) fluorescence was variably reported (Table 4).

Among Tier 1 studies, only one comparative cohort described routine ICG use for real-time perfusion assessment [12]. The remaining comparative publications did not report fluorescence integration [13,14]. In Tier 2 and Tier 3 publications, ICG was reported in multiple studies [11,15,19,20,24,25,26]. One case series noted that fluorescence imaging was unavailable on the robotic platform used [16].

ICG was described for perfusion assessment, delineation of resection margins, and guidance of transection planes. Across all studies reporting ICG use in which timing was described, fluorescence was administered after vessel ligation and mobilisation but prior to bowel transection. In this context, ICG served as a perfusion-guided transection planning tool—confirming adequate vascularisation at the planned division sites—rather than informing the level of vascular control. One study described dual ICG assessment, administered both before bowel transection and after anastomotic creation to confirm anastomotic perfusion. No study reported whether ICG findings altered the operative plan, such as changing the level of bowel division or modifying vascular strategy. The functional role of ICG—specifically whether it has the capacity to influence resection decisions when deployed prior to or during temporary vascular clamping—therefore remains unexplored in the robotic splenic flexure literature

#### 3.5.4. Technique-Specific Approaches

Several publications described distinct technical adaptations. These included single-docking crossed-arm configuration [21], vessel skeletonisation with selective branch division [24], artery-guided segmental splenic flexure colectomy [13], selective IMV preservation [25], accessory arterial branch management [20], and preoperative CT colonography with angiographic 3D reconstruction planning [11]. Modular Hugo™ system configuration was also detailed [16].

These approaches were predominantly described in Tier 2 and Tier 3 publications and were not evaluated within comparative cohort designs.

#### 3.5.5. Summary of Platform and Technical Variation

Platform selection, docking strategy, port configuration, fluorescence integration, and vessel management techniques varied across studies. Detailed technical modifications were most frequently reported in Tier 2 and Tier 3 publications (Table 4).

## 4. Discussion

This structured scoping review demonstrates that robotic splenic flexure cancer surgery is characterised by substantial operative heterogeneity within a relatively immature evidence base. Across sixteen included studies comprising 97 reported robotic resections; only three were comparative cohort analyses, while the majority were single-case technical reports. Despite this, patterns of convergence were observed. Comparative studies consistently favoured flexure-directed segmental resection with low-tie inferior mesenteric artery preservation and division of the left colic artery. Greater variability was evident in middle colic branch management, inferior mesenteric vein control, reconstruction technique, fluorescence integration, and docking configuration. Terminology relating to complete mesocolic excision and D2/D3 lymphadenectomy was inconsistently applied and rarely anatomically defined, limiting cross-study comparability. These findings suggest that technical evolution is currently outpacing standardised operative reporting.

The optimal extent of resection for splenic flexure cancer has historically been debated, with extended hemicolectomy, left colectomy, and segmental resection all advocated. Contemporary meta-analyses suggest that, when adequate lymphadenectomy is achieved, segmental resection provides comparable oncologic outcomes for isolated splenic flexure tumours [1,5,27,28]. Within the robotic literature synthesised in this review, comparative cohort studies uniformly adopted a flexure-directed segmental approach, and no study advocated routine extended hemicolectomy. This suggests that robotic surgery has not altered macro-anatomical resection philosophy but instead facilitates more precise vessel-level tailoring within a segmental framework.

The vascular ligation strategy represented the most important domain of operative heterogeneity. Comparative cohorts consistently adopted a low-tie inferior mesenteric artery strategy with division of the left colic artery and preservation of the superior rectal artery. This uniformity is notable, given that robotic platforms facilitate high central ligation, suggesting that IMA preservation represents a deliberate perfusion-preserving philosophy rather than a technical limitation. In contrast to classical CME concepts emphasising central vascular control [4] and their laparoscopic application to splenic flexure cancer [7], the robotic literature reflects a pragmatic segmental approach.

Analysis demonstrated that there was greater variability observed in middle colic branch management and inferior mesenteric vein control. Trunk versus branch ligation was often incompletely specified, and IMV division ranged from the inferior pancreatic border to more proximal control. These variations may influence perfusion and lymphadenectomy boundaries but were rarely contextualised within a defined oncologic framework. Although several studies referenced complete mesocolic excision or D3 lymphadenectomy, anatomical definitions of central vascular ligation were seldom provided. Given established lymphatic mapping data demonstrating variable nodal drainage patterns at the splenic flexure [8], inconsistent CME/D3 terminology limits meaningful comparison and interpretation of oncologic radicality. As originally defined by Hohenberger et al., a true D3 dissection at the splenic flexure requires central ligation of both the LCA at its IMA origin and the left branch of the MCA at the MCA trunk, with complete clearance of the nodal envelope surrounding both vessels—a particularly important consideration given the watershed lymphatic drainage of this region along both the IMA and MCA axes. In the included literature, however, LCA division was reported consistently while MCA branch management was documented in fewer than half of the studies, and IMA trunk preservation—though oncologically rational for perfusion—was rarely accompanied by a description of peri-IMA nodal clearance sufficient to confirm D3 adequacy. This gap between the label applied and the procedure actually performed reinforces the need for anatomically explicit operative reporting in future studies.

Interestingly, the reconstruction strategy demonstrated divergence between comparative cohorts and technical publications. Extracorporeal anastomosis predominated in comparative studies, whereas intracorporeal reconstruction was more frequently described in technical series, suggesting that adoption of intracorporeal techniques may be progressing ahead of formal comparative evaluation. Robotic systems facilitate intracorporeal suturing and stapling through enhanced articulation and three-dimensional visualisation [10], which may explain their preferential use in technical reports. Anastomotic configuration and orientation varied substantially, including side-to-side, side-to-end, functional end-to-end, and handsewn end-to-end techniques. Extraction site selection similarly varied and reflected differences in intracorporeal versus extracorporeal reconstruction, highlighting the absence of a standardised reconstructive template.

Disappointingly, fluorescence-guided perfusion assessment using indocyanine green was inconsistently reported. Given evidence that ICG fluorescence angiography reduces anastomotic leak rates in colorectal surgery, inconsistent adoption complicates comparison of reconstructive outcomes [29,30].

Platform utilisation and technical configuration further illustrated the absence of a unified operative template. Multiple robotic platforms and generations were reported with varied docking strategies and frequent under-reporting of technical detail. Robotic colorectal platforms have evolved substantially over the past decade, with successive generations associated with differing port placement philosophies and instrument capabilities [31,32]. The concentration of technical modifications within descriptive publications suggests that operative innovation is currently being disseminated primarily through technical reports rather than structured comparative evaluation. Collectively, these findings indicate that robotic splenic flexure surgery remains in a phase of exploratory technical refinement rather than protocolised standardisation.

Operative heterogeneity can be conceptualised across macro-, meso-, and micro-level domains (Table 7). At the macro level, comparative studies demonstrated convergence toward flexure-directed segmental resection. Greater divergence was observed at the meso level, encompassing arterial and venous control and lymphadenectomy classification, where identical procedural labels were often applied to materially different vascular strategies. However, at the micro level, heterogeneity was most pronounced, including variation in reconstruction technique, anastomotic configuration, extraction site, fluorescence integration, and docking strategy. While such flexibility may facilitate individualised operative execution, inconsistent reporting limits meaningful comparison.

**Figure 3:**
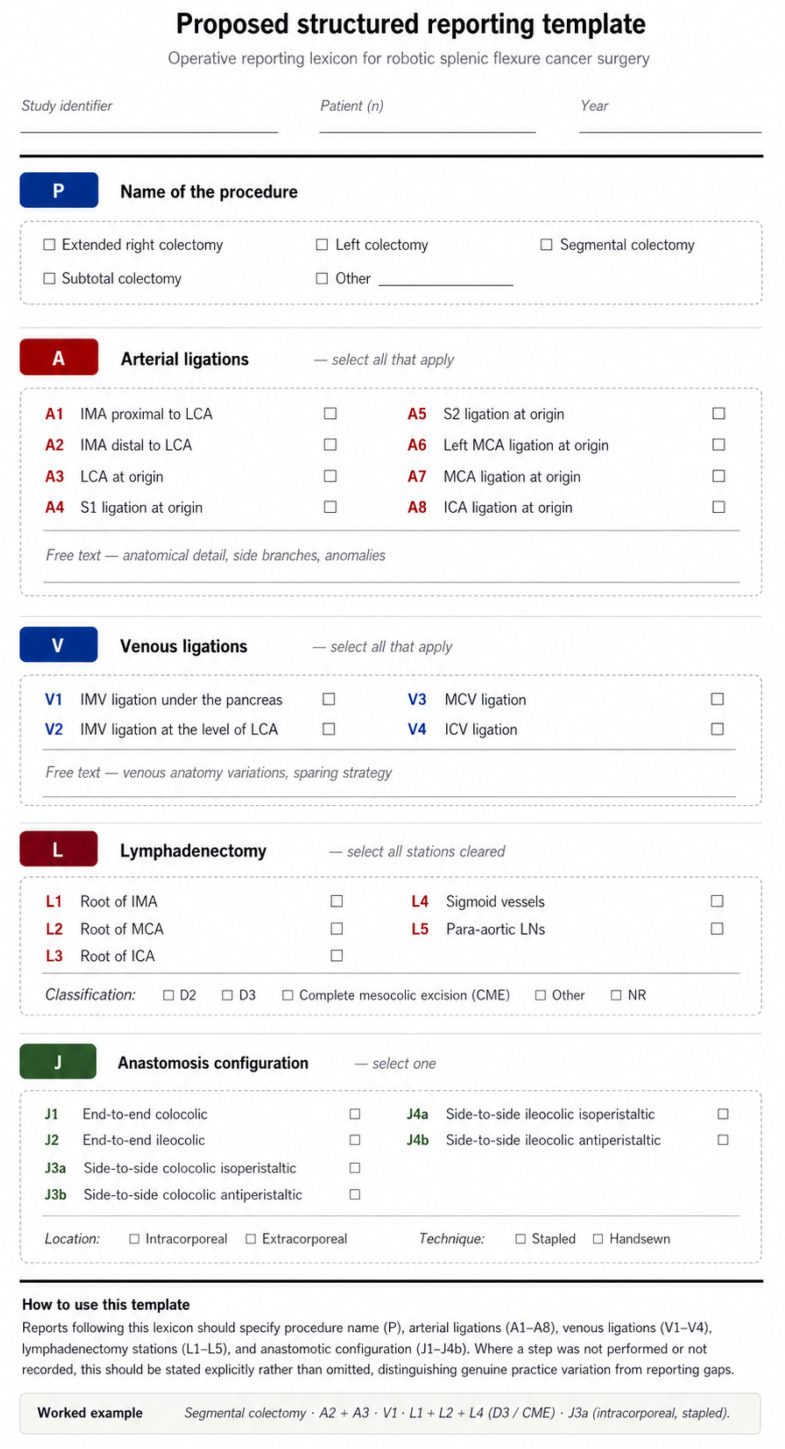
Proposed structured operative reporting template for robotic splenic flexure cancer surgery, based on the operative reporting lexicon (Table 8). The template is organised into five sections corresponding to the operative reporting lexicon: (P) name of the procedure; (A) arterial ligations, codes A1–A8; (V) venous ligations, codes V1–V4; (L) lymphadenectomy stations, codes L1–L5; and (J) anastomosis configuration, codes J1–J4b, with anastomosis location and technique. Authors are encouraged to complete each field; where a step was not performed or not recorded, this should be stated explicitly rather than omitted, in order to distinguish genuine practice variation from reporting gaps.

Collectively, these findings suggest that robotic splenic flexure surgery is characterised by macro-level convergence but meso- and micro-level technical divergence. Adoption of structured reporting aligned with these domains (Table 7) may enhance anatomical transparency and enable more rigorous comparative evaluation in future studies. The macro–meso–micro framework summarised in Table 7 has been rendered as a visual schema in Figure 3, which doubles as a structured reporting template. The three tiers are displayed as colour-coded sections containing the relevant operative decision domains: macro-level operative context, meso-level vascular and oncological decisions (including central IMA nodal clearance), and micro-level reconstruction and adjuncts. This dual-purpose presentation both communicates the framework and operationalises it as a practical instrument, which we encourage future authors to adopt as a reporting standard.

**Table 8:** Proposed structured operative nomenclature for splenic flexure resection.

Component	Descriptor	Meaning
**IMA strategy**	IMA-L	Low tie with IMA preservation
	IMA-H	High tie
**Left colic artery**	LCA+	Left colic divided
	LCA−	Left colic preserved
**Middle colic artery**	LMCA	Left MCA branch divided
	MCA	MCA trunk divided
**Reconstruction**	S2S	Side-to-side anastomosis
	S2E	Side-to-end anastomosis
	FEEA	Functional end-to-end
**Example**	IMA-L:LCA+:LMCA:S2S	Low tie IMA, LCA divided, LMCA divided, side-to-side reconstruction

## 5. Future Research Directions

The present findings suggest that technical standardisation must precede comparative evaluation in robotic splenic flexure surgery. While macro-level resection philosophy appears to be converging, persistent meso- and micro-level variability in vascular control and reconstruction limits the interpretability of existing outcome data.

Future studies should therefore prioritise structured operative reporting aligned with the macro–meso–micro framework (Table 7), incorporating explicit anatomical definitions of arterial and venous ligation, lymphadenectomy classification, and reconstructive configuration. The structured nomenclature proposed in Table 8 may provide a practical framework for consistent reporting in future studies.

In particular, future studies should explicitly report whether central lymphadenectomy surrounding the IMA was performed, irrespective of whether the IMA trunk was preserved or ligated. The robotic platform, with its enhanced three-dimensional visualisation and wristed instrumentation, is particularly well suited to precise skeletonisation of the IMA and dissection of the peri-IMA nodal envelope while preserving the trunk and superior rectal artery. This technical capability should be documented and evaluated prospectively, as it may represent one of the most oncologically meaningful advantages of robotic over laparoscopic surgery in this anatomical region.

Prospective multicentre collaboration and registry-based data collection may provide the necessary granularity to permit stratified analysis by vascular and reconstructive strategy. Without such methodological consolidation, further expansion of technical variation risks outpacing meaningful evidence generation.

## 6. Limitations

This review is limited by the small and methodologically heterogeneous evidence base currently available for robotic splenic flexure cancer surgery, with most reports comprising single-case technical publications and only a minority of comparative cohorts. Operative descriptions were frequently incomplete or non-standardised across key vascular and reconstructive domains, necessitating classification based strictly on explicitly reported anatomical detail and likely underestimating true variability. Outcome reporting was inconsistent, often restricted to short-term perioperative metrics, and long-term oncologic data were variably documented, limiting contextual interpretation of technical heterogeneity. Finally, consistent with the scoping design, formal risk-of-bias assessment was not undertaken, as the objective was structured operative mapping rather than comparative effect estimation.

## 7. Conclusions

Robotic splenic flexure cancer surgery is characterised by macro-level convergence in resection philosophy but substantial meso- and micro-level heterogeneity in vascular control, reconstruction, and technical configuration. While segmental, flexure-directed resection with low-tie IMA preservation predominates in comparative cohorts, meaningful variability persists at the level of middle colic branch management, inferior mesenteric vein control, anastomotic strategy, and platform execution. Inconsistent anatomical definitions and reporting standards limit reproducibility and complicate interpretation of oncologic and perioperative outcomes across studies.

These findings suggest that the current phase of robotic splenic flexure surgery reflects technical expansion outpacing methodological consolidation. Adoption of a structured reporting framework aligned with macro-, meso-, and micro-level operative domains may enhance anatomical transparency, enable meaningful comparative evaluation, and support the maturation of evidence in this anatomically complex and surgically nuanced field.

## Figures and Tables

**Table 1:** Comparative studies of robotic resection for splenic flexure cancer.

Study	Country	Study Design	Study Period	Total *N*	Robotic SF (*n*)	Comparator (*n*)	Platform	Follow-Up & Oncologic Reporting
Kim et al. [12]	South Korea	Retrospective comparative cohort	2012–2017	71	20	Laparoscopic (53)	da Vinci Si/Xi	Mean 18 months (range 1–59); 2-year DFS reported; recurrence described
Zang et al. [13]	China	Retrospective, propensity score-matched	2015–2019	70	19	Laparoscopic (51)	da Vinci Si	30-day outcomes only; long-term oncologic follow-up not yet available
Sugishita et al. [14]	Japan	Retrospective comparative cohort	2018–2024	52	32	Laparoscopic (20)	da Vinci Xi/X	Median 31.0 months; 3-year DFS and OS reported; recurrence patterns described

**Table 2:** Robotic splenic flexure case series.

Study	Country	Study Design	Study Period	Robotic SF (*n*)	Platform	Follow-Up
Monsellato et al. [15]	Italy	Retrospective cohort	2018–2024	12	da Vinci Si (9)/Xi (3)	Median 42 months
Carannante et al. [16]	Italy	Case series	May–September 2024	3	Hugo™ RAS	Perioperative only (no long-term follow-up)

**Table 3:** Technical notes/video vignettes/single-case reports.

Study	Country	Publication Type	SF Cases (*n*)	Platform
Aghayeva et al. [17]	Turkey	Video vignette	1	da Vinci
Benlice et al. [18]	Turkey	Video vignette	1	da Vinci Xi
Carrier et al. [19]	France	Video vignette	1	da Vinci
Da Silva et al. [20]	France	Video vignette	1	da Vinci
Erozkan et al. [21]	USA	Video vignette	1	da Vinci
Iossa et al. [22]	Italy	Video vignette	1	da Vinci
Lecot et al. [11]	France	Video vignette	1	da Vinci
Milone et al. [23]	Italy	Video vignette	1	da Vinci
Monsellato et al. [24]	Italy	Video vignette	1	da Vinci
Quezada-Diaz et al. [25]	USA	Video vignette	1	da Vinci
Bourla et al. [26]	France	Technical note	NR	da Vinci

**Table 4:** Study characteristics and resection philosophy.

Study	Tier	Robotic SF *n*	Study Design	Platform	Resection Type	CME Explicit	D3 Stated
Sugishita et al. [14]	1	32	Retrospective comparative	da Vinci Xi/X	Segmental	No	Yes (D2/D3 referenced)
Zang et al. [13]	1	19	PSM comparative	da Vinci Si	Segmental (artery-guided)	No	Not reported
Kim et al. [12]	1	20	Retrospective comparative	da Vinci Si/Xi	Partial left colectomy	Yes	Not reported
Monsellato et al. [15] (Minerva Surg)	2	12	Case series	da Vinci Si (9)/Xi (3)	Segmental	No	Not reported
Carannante et al. [16]	2	3	Case series	Hugo™ RAS	Segmental	No	Not reported
Aghayeva et al. [17]	3	1	Video vignette	Not reported	Left colectomy	Yes	Not reported
Benlice et al. [18]	3	1	Video vignette	da Vinci Xi	Left colectomy	Yes	Not reported
Bourla et al. [26]	3	1	Technical note	da Vinci Xi	Segmental	No	Not reported
Carrier et al. [19]	3	1	Video vignette	da Vinci Xi	Segmental	No	Not reported
Da Silva et al. [20]	3	1	Video vignette	Not reported	Segmental	No	Not reported
Erozkan et al. [21]	3	1	Video vignette	da Vinci Xi	Segmental	No	Not reported
Iosa et al. [22]	3	1	Video vignette	Not reported	Segmental	Yes	Not reported
Lecot et al. [11]	3	1	Video vignette	Not reported	Segmental	Yes	Not reported
Milone et al. [23]	3	1	Video vignette	da Vinci Xi	Segmental	No	Not reported
Quezada-Diaz et al. [25]	3	1	Video vignette	da Vinci	Segmental	Yes	Not reported
Monsellato et al. [24] (Colorectal Dis)	3	1	Video vignette	Not reported	Segmental (vessel skeletonization focus)	No	Not reported

**Table 5:** Vascular ligation strategy.

Study/Author	Tier	Vascular Strategy	LCA	IMA	IMV	Central IMA Nodal Clearance
Sugishita et al. [14]	1	Low tie	Dissected at bifurcation	Preserved	Dissected	Yes (D3 in 34.4% of cases; descriptive steps not provided)
Zang et al. [13]	1	Low tie	Ligated	Root dissected	Dissected	Yes (No. 253 LN dissection at IMA root described)
Kim et al. [12]	1	Low tie	Excised at origin	Preserved (SRA preserved)	Transected at pancreatic border	Not specified
Monsellato et al. [15] (Minerva Surg)	2	Low tie	Cut (ascending branch preserved once)	Preserved	Sparing strategy	Yes (complete lymphadenectomy at IMA origin as discrete step)
Carannante et al. [16]	2	Not detailed	Not detailed	Not detailed	Not detailed	Not specified
Aghayeva et al. [17]	3	Low tie	Transected at origin	Preserved	High ligation	Not specified
Benlice et al. [18]	3	Low tie	Transected at origin	Preserved	Ligated	Not specified
Bourla et al. [26]	3	Low tie	Divided	Main trunk preserved	Ligated	Yes (dissection along IMA to LCA take-off prior to division)
Carrier et al. [19]	3	Low tie	Ligated at origin	Implied preserved	Ligated	Not specified
Da Silva et al. [20]	3	Low tie	Lymphadenectomy along LCA	Not detailed	Not detailed	Not specified (lymphadenectomy along LCA only)
Erozkan et al. [21]	3	High tie (implied)	Not detailed	IMA ligated	IMV ligated	Not specified
Milone et al. [23]	3	Low tie	Divided at root	Not detailed	Divided	Not specified
Quezada-Diaz et al. [25]	3	Low tie	Divided	Preserved	Dissected	Not specified
Lecot et al. [11]	3	High tie (implied)	Not detailed	Root clipping	Not detailed	Not specified
Iosa et al. [22]	3	Not detailed	Not detailed	Not detailed	Not detailed	Not specified (generic CME reference only)
Monsellato et al. [24] (Colorectal Dis—Video)	3	Low tie	Ascending branch cut	IMA lymphadenectomy	Skeletonization of IMV	Yes (lymphadenectomy of IMA alongside LCA skeletonisation)

Central IMA nodal clearance indicates whether the nodal envelope surrounding the IMA at the root of the left colic artery was explicitly described as a discrete step, independent of IMA trunk preservation or ligation. This dissection was explicitly reported in 5 of 16 studies (31%) and was not specified in the remaining 11 (69%).

**Table 6:** Reconstruction strategy.

Study/Author	Tier	Anastomosis	Configuration	Extraction	ICG Used
Sugishita et al. [14]	1	ICA 34%/ECA 62%	FEEA/Overlap	Mini-lap	No
Zang et al. [13]	1	ECA	Side-to-side	Epigastric midline	No
Kim et al. [12]	1	ECA	Side-to-end	Transverse incision	Yes
Monsellato et al. [15] (Minerva Surg)	2	ICA	Side-to-side anisoperistaltic	Pfannenstiel	Yes
Carannante et al. [16]	2	ICA	Side-to-side	Suprapubic	No
Aghayeva et al. [17]	3	ICA	Side-to-side	Suprapubic	No
Benlice et al. [18]	3	ICA	Side-to-side	NR	No
Bourla et al. [26]	3	ICA	Side-to-side	Suprapubic	Yes
Carrier et al. [19]	3	ICA	Side-to-side mechanical	Suprapubic	Yes
Da Silva et al. [20]	3	ICA	Not specified	Pfannenstiel	Yes
Erozkan et al. [21]	3	Not reported	Not reported	Not reported	No
Milone et al. [23]	3	ICA	Side-to-side isoperistaltic	Mini-Pfannenstiel	No
Quezada-Diaz et al. [25]	3	ICA	Side-to-side isoperistaltic	Pfannenstiel	Yes
Lecot et al. [11]	3	ECA	Not specified	Not specified	Yes
Iosa et al. [22]	3	ICA	End-to-end handsewn	Not specified	No
Monsellato et al. [24] (Colorectal Dis—Video)	3	ICA	Side-to-side antiperistaltic	Not reported	Yes

**Table 7:** Operative heterogeneity framework in robotic splenic flexure cancer surgery.

Level of Operative Decision-Making	Core Elements	Observed Pattern in Current Literature	Implication
**Macro-Level** (Resection Philosophy)	Anatomical extent of colectomy	Convergence toward segmental, flexure-directed resection in comparative cohorts	Broad agreement on overall resection strategy
**Meso-Level** (Vascular & Oncologic Control)	IMA level (high vs. low tie) LCA management MCA branch vs. trunk division IMV level D2/D3 classification	Low-tie IMA common, but heterogeneity in MCA and IMV control; inconsistent anatomical definition of D3	Variability at vessel level affects lymphadenectomy boundaries and reproducibility
**Micro-Level** (Reconstruction & Technical Execution)	Intracorporeal vs. extracorporeal anastomosis Anastomotic configuration and orientation Extraction site ICG use Docking and port strategy	Marked variability, particularly in Tier 2–3 publications	Technical latitude exceeds standardisation; limits cross-study comparability

## Data Availability

All data analysed in this study are included in this published article and its Supplementary Material.

## Supplementary Materials

The following supporting information can be downloaded at https://www.mdpi.com/article/10.3390/cancers18091490/s1, File S1: Search Strategy; File S2: PRISMA Checklist.
