# Supplementary material for: A Mapping of Operative Heterogeneity in Robotic Splenic Flexure Cancer Surgery, Focusing on Vascular Ligation and Reconstructive Strategy
Source: Cancers (Basel). 2026 May 6;18(9):1490. doi: 10.3390/cancers18091490 (PMC13162774; doi:10.3390/cancers18091490)
Supplement: Supplementary file 1 [file cancers-18-01490-s001.zip › File S1.pdf]

Supplementary Material S1. Search Strategy

A structured literature search was conducted to identify studies reporting robotic surgery for splenic flexure colon cancer. The search strategy incorporated controlled vocabulary (MeSH and Emtree) and free-text terms. Searches were performed without date restriction. The final search was conducted on 15 February 2026.

1. PubMed (MEDLINE)

Date searched: 15 February 2026

Results retrieved: 65

Search strategy:

("splenic flexure"[Title/Abstract])
AND
("Robotic Surgical Procedures"[Mesh]
  OR robotic[Title/Abstract]
  OR "robot-assisted"[Title/Abstract]
  OR "robotic-assisted"[Title/Abstract])
AND
("Colonic Neoplasms"[Mesh]
  OR cancer[Title/Abstract]
  OR carcinoma[Title/Abstract]
  OR adenocarcinoma[Title/Abstract])

2. Embase (Ovid)

Platform: Ovid Embase (1974 to present)

Date searched: 15 February 2026

Results retrieved: 37

Search strategy:

---

1. "splenic flexure".ti,ab.

2. robotic.ti,ab.
3. robot-assisted.ti,ab.
4. exp robotic surgery/
5. 2 OR 3 OR 4
6. exp colon cancer/
7. 1 AND 5 AND 6

Study Selection Process

All records identified from PubMed and Embase were exported in RIS format and imported into Rayyan for deduplication and screening. Titles and abstracts were screened against predefined eligibility criteria. Full texts were reviewed where necessary. Conference abstracts, reviews, letters, and studies in which splenic flexure cases could not be separated from other colonic locations were excluded during screening.
